# Isolation in Older Adults in the United States and Japan: An Early Examination of IOT Possibilities

**DOI:** 10.1093/geroni/igab046.1744

**Published:** 2021-12-17

**Authors:** Dana Bradley, Carmen Sceppa, Carmen Sceppa

## Abstract

Isolation in older adults is a growing problem in both the US and Japan. This symposium showcases work funded by NSF and JST (Japan Science Technology Committee) to develop smart technology to create caring, connected communities by integrating gerontology and technology research. The U.S. and Japan are experiencing dramatic population aging and share several similarities: Populous (U.S. 327 million at #3 and Japan 127 million at #11) and economically developed (GDP: the U.S. #1 and Japan #3) and isolation was increasing even before the pandemic. This multi-year project addresses the challenges of isolation by using smart technologies in culturally appropriate ways to support older adults and suggests ways that isolation and loneliness may be managed by older persons, local governments, and NGOs. The first paper examines the experiences of loneliness as characterized by early retirees in both countries. The interdisciplinary research team has used this qualitative set of case studies to identify promising technology support points. Our second paper explores these issues surrounding isolation using data from Study on the Lifestyle and Values of Senior Citizens (Japan). This analysis focuses on longitudinal data from both countries and helps situate our work outside the Covid-19 Pandemic. The third presentation focuses on the intersection between culture and technology and proposes a continued research collaboration model. Our discussion will highlight how community stakeholders in U.S. and Japan have a role in creating evidence-based adaptive environments to detect and mitigate isolation by developing and using gero-centric approaches.

